# Early Potent Protection against Heterologous SIVsmE660 Challenge Following Live Attenuated SIV Vaccination in Mauritian Cynomolgus Macaques

**DOI:** 10.1371/journal.pone.0023092

**Published:** 2011-08-10

**Authors:** Neil Berry, Claire Ham, Edward T. Mee, Nicola J. Rose, Giada Mattiuzzo, Adrian Jenkins, Mark Page, William Elsley, Mark Robinson, Deborah Smith, Deborah Ferguson, Greg Towers, Neil Almond, Richard Stebbings

**Affiliations:** 1 Division of Retrovirology, National Institute for Biological Standards and Control (NIBSC), Health Protection Agency (HPA), South Mimms, Potters Bar, Hertfordshire, United Kingdom; 2 Division of Biotherapeutics, NIBSC/HPA, South Mimms, Potters Bar, Hertfordshire, United Kingdom; 3 MRC Centre for Medical Molecular Virology, Division of Infection and Immunity, University College London, London, United Kingdom; University of Sao Paulo, Brazil

## Abstract

**Background:**

Live attenuated simian immunodeficiency virus (SIV) vaccines represent the most effective means of vaccinating macaques against pathogenic SIV challenge. However, thus far, protection has been demonstrated to be more effective against homologous than heterologous strains. Immune correlates of vaccine-induced protection have also been difficult to identify, particularly those measurable in the peripheral circulation.

**Methodology/Principal Findings:**

Here we describe potent protection in 6 out of 8 Mauritian-derived cynomolgus macaques (MCM) against heterologous virus challenge with the pathogenic, uncloned SIVsmE660 viral stock following vaccination with live attenuated SIVmac251/C8. MCM provided a characterised host genetic background with limited Major Histocompatibility Complex (MHC) and TRIM5α allelic diversity. Early protection, observed as soon as 3 weeks post-vaccination, was comparable to that of 20 weeks vaccination. Recrudescence of vaccine virus was most pronounced in breakthrough cases where simultaneous identification of vaccine and challenge viruses by virus-specific PCR was indicative of active co-infection. Persistence of the vaccine virus in a range of lymphoid tissues was typified by a consistent level of SIV RNA positive cells in protected vaccinates. However, no association between MHC class I /II haplotype or TRIM5α polymorphism and study outcome was identified.

**Conclusion/Significance:**

This SIV vaccine study, conducted in MHC-characterised MCM, demonstrated potent protection against the pathogenic, heterologous SIVsmE660 challenge stock after only 3 weeks vaccination. This level of protection against this viral stock by intravenous challenge has not been hitherto observed. The mechanism(s) of protection by vaccination with live attenuated SIV must account for the heterologous and early protection data described in this study, including those which relate to the innate immune system.

## Introduction

The development of safe, effective vaccination strategies to control the HIV/AIDS pandemic remains an important goal for global human health, although significant obstacles to achieving this aim remain following disappointing results from recent Phase II/III clinical HIV vaccine trials [1]. Candidate HIV vaccine design is further compounded by the diverse sequence variation which characterises the worldwide spread of HIV, represented by multiple HIV-1 groups (M, N and O), further divided into multiple subtypes or clades and complex recombinant forms [2], [3]. Ideally, vaccination would prevent infection completely or reduce onward virus transmission, although the appropriate responses needed to be induced by an effective HIV vaccine strategy to prevent infection remain unclear. Vaccination with live attenuated SIV vaccines in the SIV/macaque model have consistently demonstrated potent vaccine protection from wild-type virus challenge [4] either to protect completely from detectable infection, or reduce markedly the replication of the challenge virus administered by either the intravenous or mucosal routes [5]–[30]. Yet even within these model systems discrepancies exist regarding the outcome of vaccine/challenge studies using this vaccine approach. In particular, there is uncertainty as to the potency of vaccine protection against heterologous virus challenge.

Although the use of live attenuated retroviruses as vaccines suitable for human use is precluded on safety grounds [31], [32], [33], with both reversion of the attenuated virus vaccine to wild-type [28] and recombination with challenge virus [18], [34] having been described, the identification and reproduction of protective vaccine responses by safer means remains an important goal of HIV vaccine research. While the outcome of live attenuated vaccine studies may be dependent on different variables such as the vaccine strain and duration of vaccination, the challenge virus and its biological properties in vivo and the host species, analysis of these variables and their influence on study outcomes provides the opportunity to identify processes by which this vaccination approach protects.

We have been characterising the protection conferred by a nef-disrupted viral clone derived from SIVmac251/32H, designated SIVmacC8 [35]. In previous vaccine studies we have demonstrated the ability of SIVmacC8 to protect from both a moderately replicating, cloned virus challenge (SIVmac32H/J5) [7], [24], [25] and a vigorously replicating, uncloned homologous challenge stock (SIVmac251/32H/L28) [8]. While protection has been observed as early as 21 days post-vaccination against SIVmac251/J5 [24], [25], protection is superior after longer periods of vaccination, up to 20 weeks, particularly against the SIVmac251/32H/L28 stock [8]. Although protection conferred by SIVmacC8 against SIVmacJ5 coincides with the appearance of detectable CD8+ T cell responses [24] it does not appear to be abrogated by profound CD8+ T cell depletion [25], nor can protection be transferred by immune serum [36]. Despite having different biological properties in vivo, both virus challenge stocks in these studies were genetically homologous to the SIVmac251/C8 vaccine strain.

Therefore, to extend these studies, the breadth of vaccine protection conferred by SIVmacC8 was assessed by challenging with an antigenically and genetically distinct virus stock. The uncloned SIVsmE660 virus stock has been used in studies of Indian rhesus macaques (Macaca mulatta) vaccinated with SIVmac239Δ3 [29] and SIVmac239Δnef [18] vaccines. SIVsmE660 is considered to represent a genetically diverse, heterologous virus challenge stock containing multiple sequences in a viral swarm having undergone only minimal passage in rhesus macaques (RM).

Recent developments in the immunogenetic characterisation of Mauritian cynomolgus macaques (MCM), which express limited genetic diversity, have identified their valuable place to study the role of host MHC genetics in immunogenicity and vaccine studies [37]–[45]. Only 7 MHC haplotypes encompassing Class IA, IB and MHC Class II DR, DP and DQ regions, are present in MCM at frequencies >1% [37], [38]. The relative ease of MHC haplotype characterisation and high frequency of selected MHC haplotypes have enabled small retrospective population studies to be performed. Statistically robust associations between M3 or M6 haplotypes and superior viraemic control of SIV or SHIV viruses in naïve-challenged and vaccinated individuals have been identified [43]–[45].

Since relatively little is known about the infectivity and replication properties of SIVsmE660 in MCM, and to establish the infectious titre of this stock in this species, an in vivo titration was performed. Subsequently, a vaccine study was conducted to determine if vaccination of Mauritian-derived cynomolgus macaques with SIVmacC8 could protect against SIVsmE660 challenge. The outcome of the vaccine study was interpreted in the knowledge of the replication dynamics of SIVsmE660 in unvaccinated MCM and the potential influence of MCM MHC genetics and TRIM5α polymorphism on vaccine protection in this system. In keeping with previous studies [24], [25], [8], we compared the effects of vaccination with SIVmacC8 for 3 and 20 weeks prior to SIVsmE660 challenge.

Here we demonstrate that vaccination of naive MCM with SIVmacC8 prevents detectable infection against challenge with the heterologous, pathogenic SIVsmE660 challenge stock in a high proportion of vaccinates, irrespective of the MHC genetic background of the host. Potent vaccine protection was established as early as three weeks with no difference between 3 and 20 week vaccine regimens. Understanding how such early protection is generated with live attenuated SIV in this model system will inform HIV/AIDS vaccine design.

## Results

### Infectivity and RNA kinetics of the SIVsmE660 challenge stock in MCM

**Figure 1:**
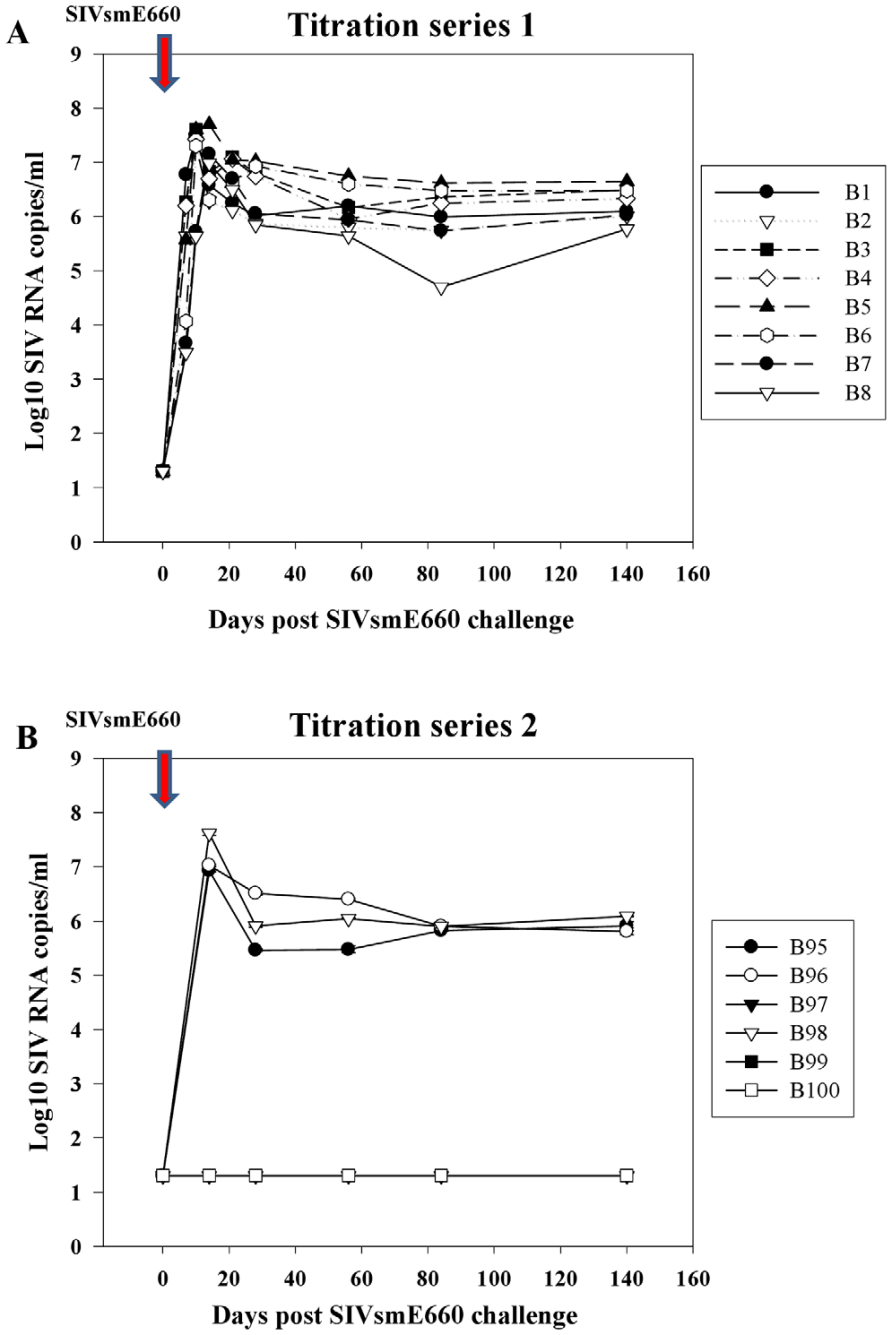
Infectivity of SIVsmE660 challenge stock in Mauritian cynomolgus macaques. Levels of plasma SIV RNA measured by qRT-PCR with R/U5 primers following multiple SIVsmE660 challenges in an in vivo titration of unvaccinated Mauritian cynomolgus macaques. Red arrow shows point of SIVsmE660 inoculation (day 0). Plasma SIV RNA levels are shown for individual macaques (B1–B8) and (B95–B100) in two independent titration series (panels A and B respectively) comparing acute, peak and steady-state chronic phases of virus replication post-SIVsmE660 challenge. Outcome of administering high dose (low dilution) viral inocula for pairs of macaques receiving a 1/10 (Group 1 ; B1, B2), 1/100 (Group 2; B3, B4); 1/1000, (Group 3, B5, B6), and 1/10,000 (Group 4, B7, B8) dilution of virus are shown in panel A. Plasma viraemia for the second dilution series at 1/10,000 (Group 5; B95, B96), 1/100,000 (Group 6; B97, B98) and 1/1,000,000 (Group 7, B99, B100) dilutions are shown in panel B. No evidence of plasma virus was detected in B97, B99 and B100 at any time point.

The infectivity and in vivo titre of the SIVsmE660 virus stock in Mauritian cynomologus macaques was first determined (Figure 1). In an initial titration series groups 1–4, (B1–B8; 1/10–1/100,000 dilutions), productive infection was established in all macaques as determined by viral RNA (vRNA) analysis and virus co-culture (Figure 1A; Table S1), although no end-point was reached. Plasma vRNA peaked in B1-B6 at day 10 but was slightly delayed (∼day 14) in B7 and B8. In a second series, groups 5–7 (B95–B100; 1/10,000–1/1,000,000 dilution; Figure 1B), productive infection was established in 2/2 inoculated with 1/10,000 dilution (B95, B96), 1/2 with 1/100,000 dilution (B98) and 0/2 with 1/1,000,000 dilution (Figure 1B, Table S1). Virus replication kinetics in B95, B96, B98 were very similar to each other and challenges with lower virus dilutions; B97, B99 and B100 remained vRNA undetectable with no evidence of transient or late infection.

Of 11 naïve macaques productively infected with SIVsmE660, mean day 14 and 84 viraemia levels were 6.91±0.14 log_10_ and 5.95±0.16 log_10_ SIV RNA copies/ml respectively, demonstrating high, reproducible infectivity and replication potential of the SIVsmE660 challenge stock in MCM. Viral RNA kinetics in productively infected individuals were virtually identical irrespective of challenge dose (Figure 1), the second highest vRNA titre at day 14 (B98; 7.62 log_10_ SIV RNA copies/ml), challenged with 1/100,000 virus dilution. Titration results indicate a MID_50_ for the SIVsmE660 challenge stock in MCM to be represented by 1/100,000 dilution of the initial virus stock. Hence, 10 MID_50_ (1/10,000 dilution of the original SIVsmE660 stock) was used in the subsequent vaccine study.

### MHC haplotype frequency in MCM

**Figure 2:**
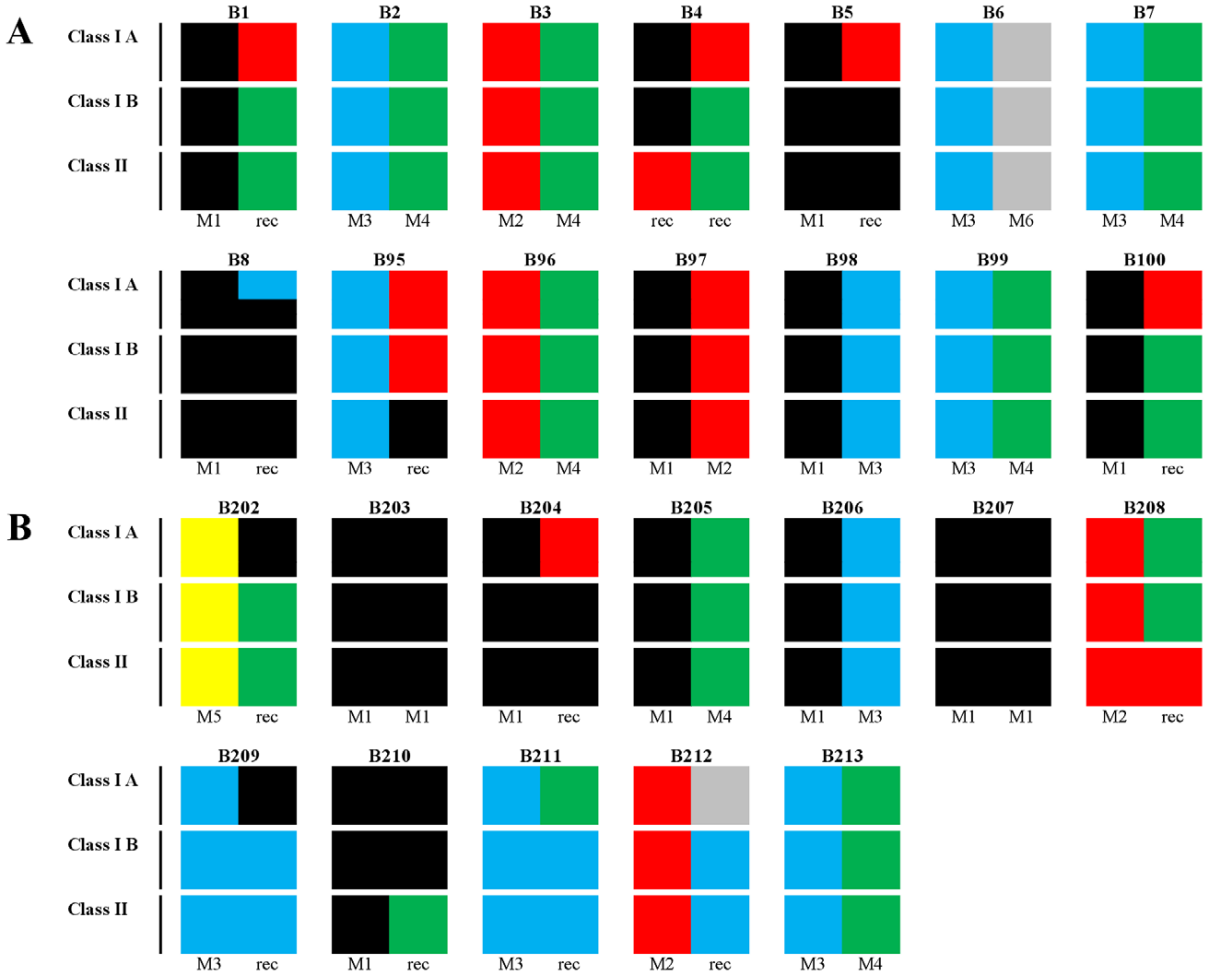
Distribution of MHC haplotypes in 26 Mauritian cynomolgus macaques. MHC genotype distribution for the in vivo titration of SIVsmE660 (see Figure 1) in macaques B1–B8 and B95–B100 (panel A). Twelve macaques (B202–B213) were included in the vaccine/challenge study (panel B). Distribution of M1–M6 haplotypes for Class IA, Class IB and Class II for each animal is shown in pictorial format represented as: M1 (black); M2 (red); M3 (blue), M4 (green), M5 (yellow) and M6 (grey). No M7 haplotype was identified. Recombinants are represented as multiple colours.

Host MHC immunogenetic background of all 26 MCMs is presented pictorially in Figure 2. All major previously described MCM haplotypes (M1–M6) for MHC Class IA, Class IB and Class II regions were represented in either the MCM in the in vivo titration (B1–B8; B95–B100; Figure 2A) or vaccine study (B202–B213, Figure 2B). No relationship between the ability of SIVsmE660 to replicate in vivo in naive MCM and host MHC haplotype frequency was identified. High levels of peak and persisting plasma vRNA loads in productively infected naive MCM challenged with SIVsmE660 occurred irrespective of MHC haplotype combination, including recombinants (Figure 1). The slight delay in the vRNA peak at 14 days in B7 and B8 challenged with 10 MID_50_ of virus compared with B1–B6 where the peak was 10 days post infection, yet B7 had an identical MHC profile to B2 (haplotypes M3/M4). Hence, among naive MCM, MHC haplotype did not appear to affect peak or persisting viral loads, though robust statistical analyses were confounded by low frequency of haplotypes M5 and M6 in the study cohort.

### TRIM5α polymorphism

The TRIM5α/TRIM-Cyp genotype was determined for all 26 MCM in this study (Table 1). Only 3 distinct TRIM5α genotypes were identified. The Mamu4 genotype [46] was present in all 26 macaques, most commonly as a homozygous genotype in 14 out of 26 (54%) MCM or as heterozygous 4/8 or 4/9 genotype in 6 out of 26 (23%) respectively. TRIM5α alleles were relatively evenly distributed between both the titration and vaccine studies (Table 1). No TRIM-Cyp variants were identified. These data concur with other unpublished findings (NB, GJT, NR). Although only represented by three TRIM5α variants, in unvaccinated macaques there appeared to be no confounding influence of TRIM5α polymorphism on SIVsmE660 replication in this study population, with all unvaccinated macaques challenged with SIVsmE660 displaying very similar plasma viral RNA kinetics *in vivo*.

**Table 1:** Frequency and distribution of TRIM5α alleles in unvaccinated controls and SIVmacC8 vaccinates challenged with SIVsmE660.

Group	Allele4	Allele4/8	Allele4/9
**Controls**	B5	B1	B2
	B7	B3	B4
	B95	B100	B6
	B96	B210	B8
	B98	B211	B97
	B99	(n = 5)	(n = 5)
	B212		
	B213		
	(n = 8)		
**Vaccinates**	B202	B207	B205
	B203	(n = 1)	(n = 1)
	B204		
	B206		
	B208		
	B209		
	(n = 6)		

The number of macaques in each group is indicated (n).

### SIVmacC8 vaccine kinetics

**Figure 3:**
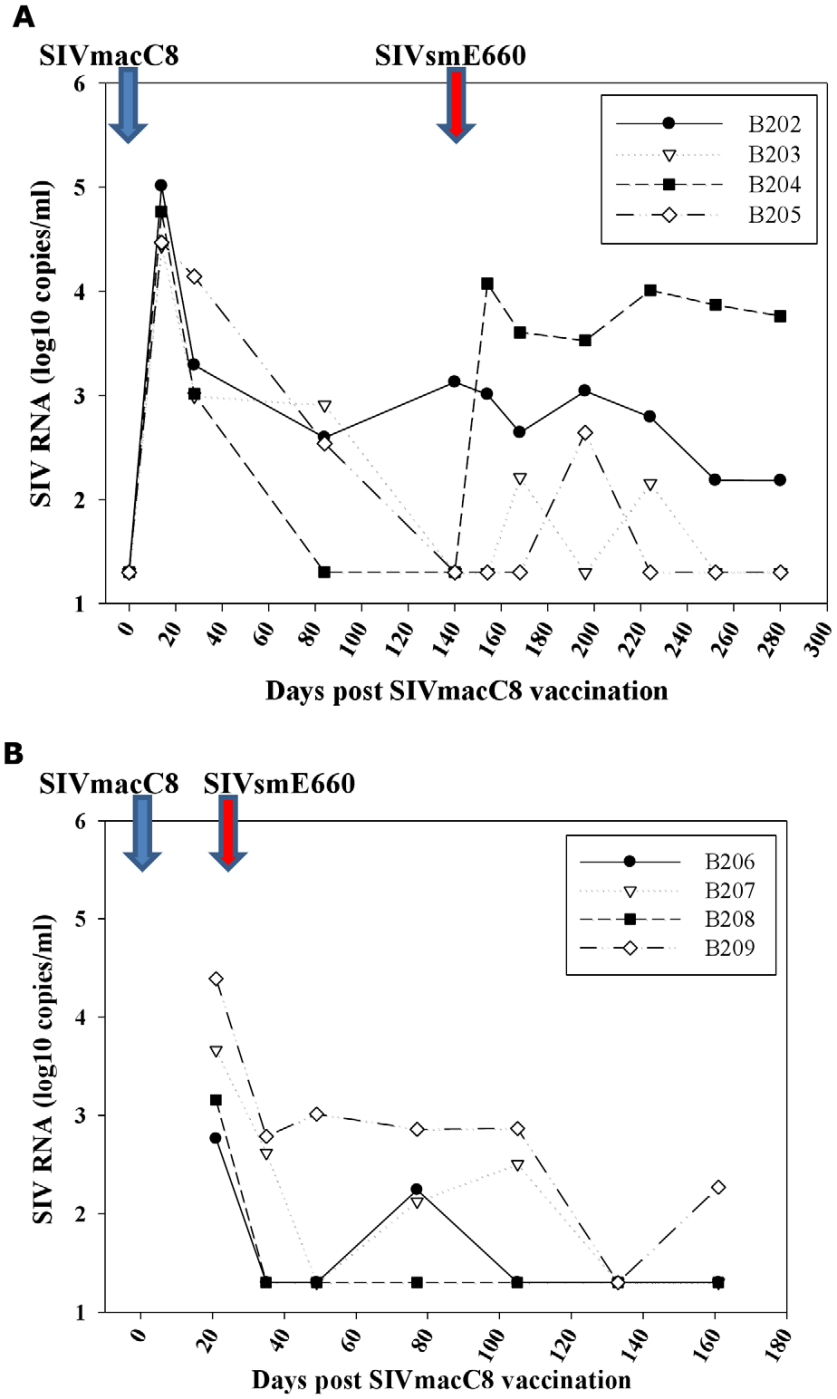
Vaccine virus kinetics. Kinetics of virus replication post-SIVmacC8 vaccination measured by SIVmac251/C8-specific quantitative RT-PCR in 20 weeks (Group A) and 3 weeks (Group B) vaccinates. Blue arrow shows point of SIVmacC8 inoculation, red arrow point of SIVsmE660 challenge. Differences in vaccine kinetics between vaccinates in Panel A, Group A (B202–B205), after the peak of virus replication was seen, prior to SIVsmE660 challenge. Panel B, shows a single time-point 21 days post SIVmacC8 inoculation, ie day of SIVsmE660 challenge. In both Groups A and B, recrudescence of the vaccine virus SIVmacC8 upon SIVsmE660 challenge is shown.

Eight MCM were inoculated by intravenous injection with 5000 TCID_50_ SIVmacC8. Plasma SIV RNA levels were monitored by a quantitative real-time gag PCR assay (Groups A and B; Figure 3). All eight vaccinates were productively infected with SIVmacC8. Group A, vaccinated for 20 weeks (Fig 3A), all displayed similar levels of plasma viraemia at 14 days post-inoculation (4–5 log_10_ SIV RNA copies/ml), comparable with historical data with this virus vaccine [8], [47]. Analysis of vaccine virus kinetics over the 20 week vaccination period indicated one of three steady-state vRNA profiles: i) enhanced control of SIVmacC8 viraemia (B204) to undetectable levels at time of SIVsmE660 challenge ii) typical reductions in viraemia from the peak declining to similar levels (B203, B205), returning to baseline levels, iii) low, persisting vRNA levels (B202) at ∼3 log_10_ SIV RNA copies/ml. At time of SIVsmE660 challenge SIV RNA levels were ≤10^3^ copies/ml in all vaccinates.

Differences in vRNA levels in 20 week vaccinates (Group A, B204–B205) also appeared unrelated to MHC haplotype composition (Figure 2B). All were positive for haplotype M1 in the Class IA region. Group B (Figure 3B), vaccinated with SIVmacC8 for three weeks all had quantifiable viraemia at time of SIVsmE660 challenge at levels also consistent with previous SIVmacC8 vaccinations.

### Antibody levels post SIVmacC8 vaccination

**Figure 4:**
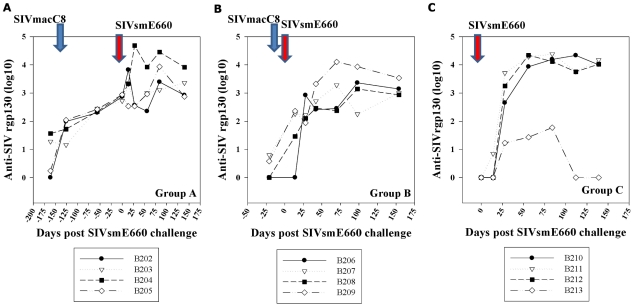
Anti-gp130 levels. Anti-gp130 levels post-SIVmacC8 vaccination and post SIVsmE660 challenge determined by binding antibody ELISA, (day 0 represents day of SIVsmE660 challenge) shown for Groups A, B and C. Group A shows anti-SIVmacC8 gp130 levels for 20 weeks prior to SIVsmE660 challenge (at day 0) with an anamnestic response detected in B204. Panel B shows anti-gp130 responses in Group B (3 week vaccinates) and Group C (SIVsmE660 naïve challenge controls) respectively.

All 20 week SIVmacC8 vaccinates (Group A) exhibited similar binding antibody profiles against rgp130 (Figure 4A, B), typical for this vaccine. At day of SIVsmE660 challenge, anti-SIVrgp130 levels were ∼2.5 log_10_ in all 20 week vaccinates. Anti-gp130 titres were more variable among three week vaccinates (Group B), with no response detected in B206 prior to SIVsmE660 challenge (Figure 4B). Neutralising antibody titres were assessed retrospectively with SIVmac251/J5, representing the vaccine virus and SIVsmE660 the challenge virus (Table 2). On the day of SIVsmE660 challenge neutralising antibody titres were undetectable (<1.0 log_10_) for both Groups A and B, against SIVsmE660. For SIVmac251/J5, neutralisation titres were detected in all 20 week vaccinates (Group A), although at low levels (2.13, 2.20, 1.90 and 1.98 log_10_ for B202–B205 respectively) but were undetectable (<1.0 log_10_) in 3 week vaccinates (Group B).

**Table 2:** Neutralising antibody titres on day of SIVsmE660 challenge.

	ID	SIVmac251	SIVsmE660
Group A	B202	2.13	<1.0
	B203	2.20	<1.0
	B204	1.90	<1.0
	B205	1.98	<1.0
Group B	B206	<1.0	<1.0
	B207	<1.0	<1.0
	B208	<1.0	<1.0
	B209	<1.0	<1.0

Log_10_ neutralising antibody titres on day of SIVsmE660 challenge are shown for Group A and Group B vaccinated for 20 and 3 weeks respectively with SIVmacC8. <1.0 log_10_ represents undetectable levels. ID  =  macaque identity.

### CD4 lymphocyte counts

**Figure 5:**
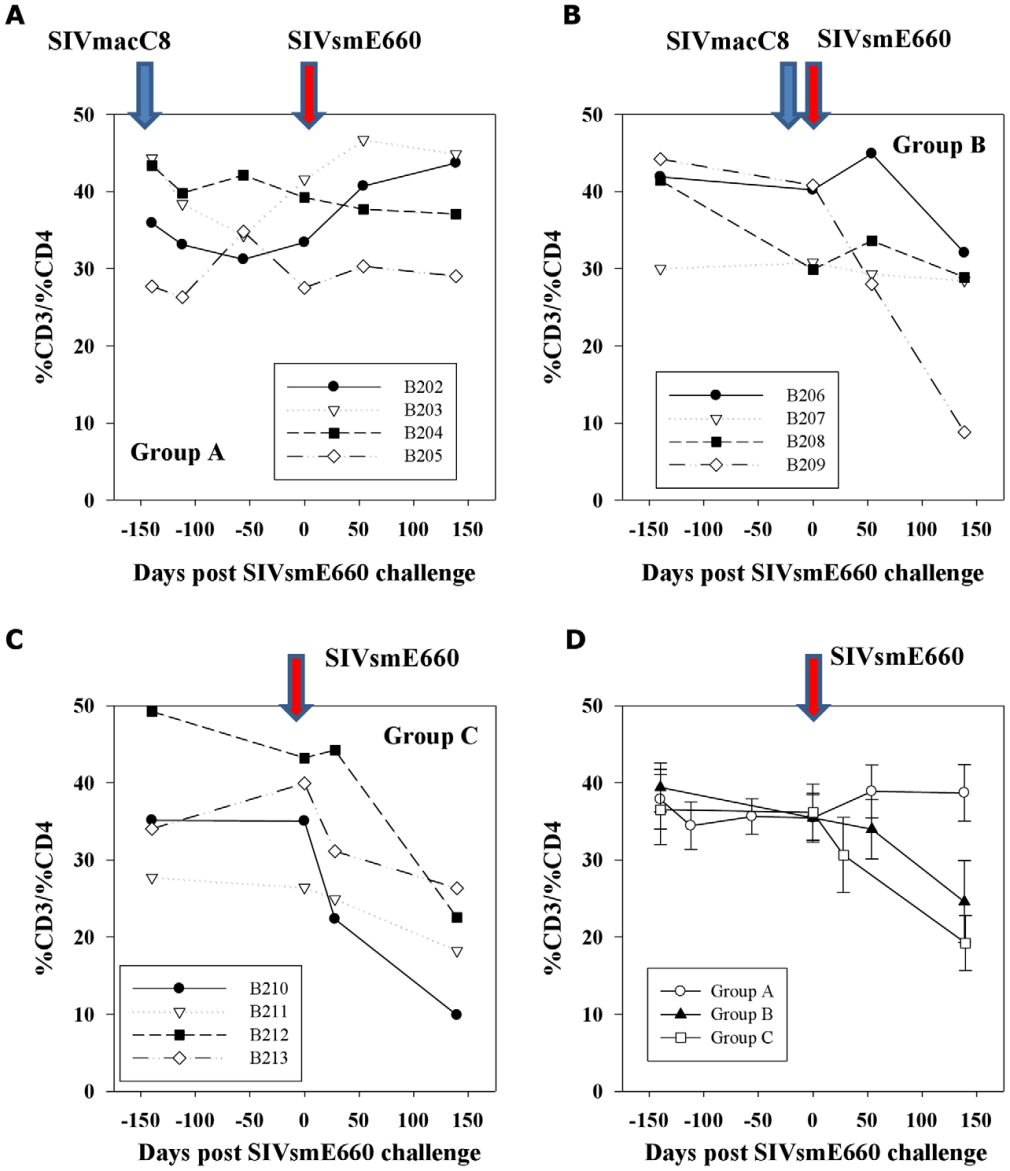
CD4 lymphocyte percentages. CD4 lymphocyte percentages are shown for 20 week SIVmacC8 vaccinates (Group A; panel A) or 3 weeks (Group B; panel B), following SIVsmE660 challenge and unvaccinated challenge controls (Group C; panel C). Timings of all bleeds were taken from initial time of SIVsmE660 challenge (day 0). Group mean values with SE of the mean values are shown in panel D.

CD4 lymphocytes were monitored during vaccination and challenge periods (Figure 5). Prior to SIVmacC8 inoculation, mean percentages of CD4+ lymphocytes in all MCM were within the 30–40% reference range. In 335 naïve cynomolgus macaques analysed retrospectively this was 35.9±7.3 (SD) where the CD4+ T cell range±2SD was 21.4–50.4% representing a 95% distribution of the data. Significant immunological abnormalities were considered to have occurred when sustained CD4 cells fell below 21%. SIVsmE660 infection of naive MCM (Group C) led to an overall reduction in circulating CD4+ lymphocyte percentages (Figure 5C) which were preserved in Group A vaccinates. Individual CD4 percentages (Figure 5A–C) displayed a wide variation though there were distinct trends, with CD4 counts in all Group A vaccinates being well preserved irrespective of the challenge outcome (Figure 5A). Group B showed more variation, in particular B209 which exhibited a precipitous drop in CD4 counts after 20 weeks SIVsmE660 infection (Figure 5B), comparable to unvaccinated challenge controls (Figure 5C). Interestingly, CD4 counts appeared to be declining prior to SIVsmE660 challenge in this macaque. Individual values were reflected in the overall group mean data (Figure 5D).

### Total levels of plasma SIV RNA post-SIVsmE660 challenge

**Figure 6:**
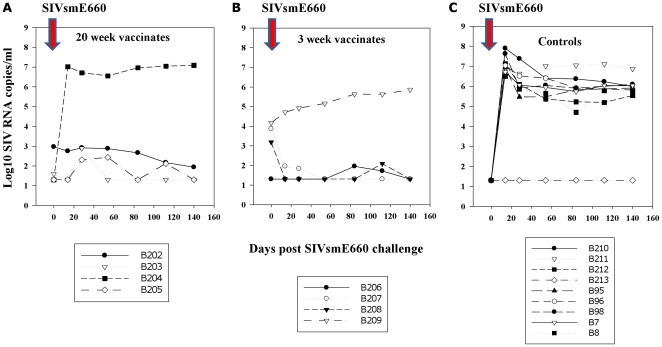
Total SIV RNA levels in plasma. SIV RNA levels, expressed as log_10_ SIV RNA copies/ml, determined by virus-common qRT-PCR assay based on conserved R/U5 sequences in genomic SIV RNA. Panel A, 20 weeks vaccinates (Group A; B202–B205); panel B, 3 weeks vaccinates (Group B; B206–B209); panel C, 9 naive controls challenged with low dose (1–10MID_50_) SIVsmE660.

Although similar vRNA kinetics between different naive MCM challenged with widely ranging doses of SIVsmE660 were noted (Figure 1), comparisons of outcome of vaccinates were compared with unvaccinated controls that received 10 or 1 MID_50_ virus only representing the most biologically relevant challenge doses. Figure 6 shows total plasma SIV RNA levels, measured by real-time R/U5 qRT-PCR, for 20 and 3 week vaccine groups (Figure 6A,B respectively) and nine productively infected SIVsmE660 control macaques receiving 1–10 MID_50_ SIVsmE660 (Figure 6C). Acute plasma vRNA levels in controls were 6.62±0.4 log_10_ SIV RNA copies/ml (day 14) and steady-state levels of 5.69±0.35 log_10_ SIV RNA copies/ml (day 84).

With the exception of B204 (Group A) vaccinated for 20 weeks, all other vaccinates had marked reductions in acute SIV RNA levels 14 days post SIVsmE660 challenge compared with naive challenge controls (Figure 6C). By this overall marker of virus replication a strong vaccine effect in acute and chronic control of SIVsmE660 challenge detectable in peripheral blood in both 20 and 3 week vaccinates, compared with naive challenge controls, was observed.

**Figure 7:**
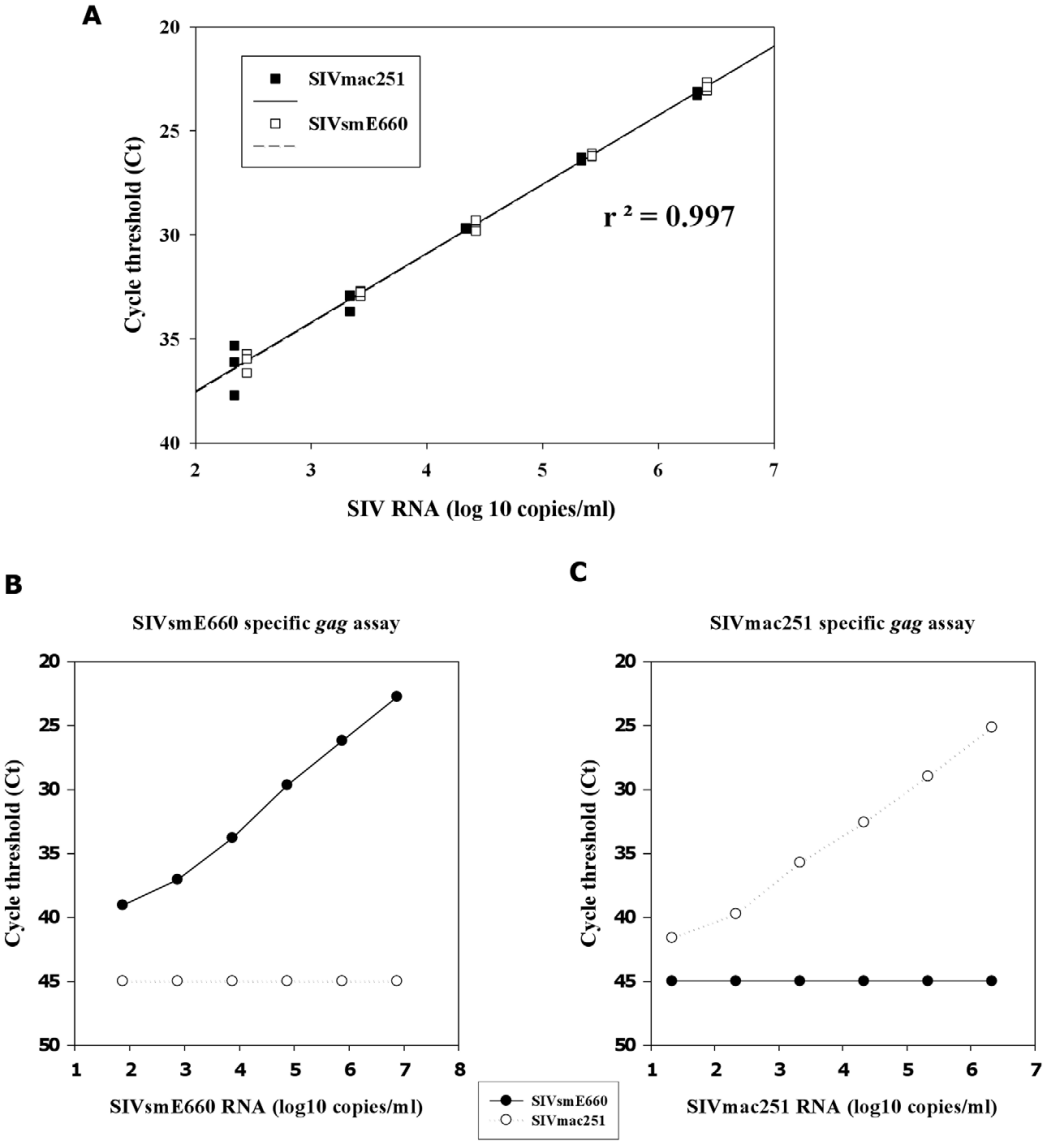
Type common and type specific PCR analysis of SIVmac251 and SIVsmE660. Panel A shows equivalent amplification of SIVmac251 and SIVsmE660 with R/U5 primers targeting the 5′ region of genomic SIV RNA. Comparable regression curves were obtained with a high titre SIVmac251 reference panel [65] and a SIVsmE660 plasma pool derived from day 10 and 14 bleeds of titration macaques B1–B4. Subsequent cross-titration experiments conducted with the heterologous gag-based SIVsmE660 and SIVmacC8 plasma viral RNA quantification assays compared threshold detection levels with viral RNA in-put copy number. Assay specificity was demonstrated with the same pooled SIVsmE660 plasma from control MCMs diluted in negative plasma and a SIVmac251/L28 plasma reference panel [65] for SIVmac251 sequences. Respective assays demonstrated specific amplification of the SIVsmE660 (challenge, panel B) and SIVmac251 (vaccine, panel C) viruses by gag-specific RT-PCR across a 6 log_10_ dynamic range. Intra-assay variation between replicates of the SIVsmE660-specific assay was 0.09 SD with a minimum amplification efficiency of 94.9%. Replicates included at least three runs with a coefficient of variation of <3%. No cross-reactivity with the heterologous virus was detected above a sensitivity of detection limit of 50 SIV RNA copies/ml.

Virus replication remained under control in all vaccinates, except B204 which had higher acute and persisting vRNA loads (7.01 and 6.95 log_10_ SIV RNA copies/ml, 14 and 84 days respectively) and B209 where viraemia increased over a 20 week period in the 5–6 log_10_ range. Taking together data for the eight vaccinates and eight unvaccinated controls challenged only with 10 MID_50_ SIVsmE660, statistically highly significant reductions (95% CI) in both peak (d14; p<0.005 Mann-Whitney) and persisting (d84, p<0.005 Mann-Whitney) viraemia post SIVsmE660 challenge were observed. For individual vaccine groups, virus levels were 3.09±1.35 log_10_ and 3.05±1.33 log_10_ SIV RNA copies/ml (Group A) and 2.33±0.81 log_10_ and 2.54±1.04 log_10_ SIV RNA copies/ml (Group B) at 14 and 84 days post SIVsmE660 challenge respectively. However, a proportion of this total figure could also be due to detection of the vaccine virus, particularly in 3 week vaccinates, since the R/U5 assay was demonstrated to be efficient at detecting both SIVmac251 and SIVsmE660 sequences (Figure 7A).

### Virus-specific real-time PCR

To elucidate virus-specific responses, type-specific PCR assays were applied. Initial experiments indicated the published gag real-time PCR assay [8] while efficient at detecting SIVmac251 sequences, did not detect SIVsmE660/E543 variants. Therefore a comparable gag-based SIVsmE660-specific assay was developed to enable delineation of individual virus infection kinetics. Using sequences available in the Los Alamos sequence database, (www.lanlhiv.gov) and ∼700bp gag sequence recovered from unvaccinated SIVsmE660 challenged macaques, primer and probe sequences for specific amplification of SIVsmE660 sequences by real-time PCR were designed. Initial work-up experiments identified sequences which did not cross-react with the heterologous vaccine strain SIVmacC8 (data not shown). A SIVmac251-based RNA reference panel was used in parallel to a SIVsmE660 plasma series constructed from a pool of high titre plasma derived from unvaccinated SIVsmE660 controls from the titration experiment, diluted similarly in negative macaque plasma (Figure 7A).

Each dilution series was first compared with the R/U5 qPCR assay demonstrating equivalent regression curves (r^2^ = 0.997) across ∼6 log_10_ dynamic range, using RNA extracted from either the SIVmac251 or SIVsmE660 plasma dilution series (Figure 7A) and provided a basis to compare virus-specific RT-PCR assays. Using these independent dilution panels, the virus specificity of the two gag-based assays were determined (Figures 7B, C). These data indicate no evidence of cross-reactivity in the heterologous assay, even at high viral titres (>7 log_10_ SIV RNA levels). Both SIVsmE660 and SIVmac251-specific assays exhibited comparable detection sensitivity at 50 SIV RNA copies/ml plasma.

Comparable data were generated for quantitative DNA (qDNA) determinations, optimised on high copy number SIVsmE660 DNA diluted to an extinction end-point in herring sperm carrier DNA. A linear relationship between target input DNA and signal was demonstrated (not shown). Assay specificity was further established by cross-titration of high copy number input SIVmac251 and SIVsmE660 DNA, capable of detecting single copies of SIVsmE660 DNA in a background of 100,000 cell equivalents of genomic DNA. These assays were applied to plasma vRNA and tissue qDNA estimations of viral copy number in the vaccine study.

### Absence of SIVsmE660 RNA and DNA in protected vaccinates

**Figure 8:**
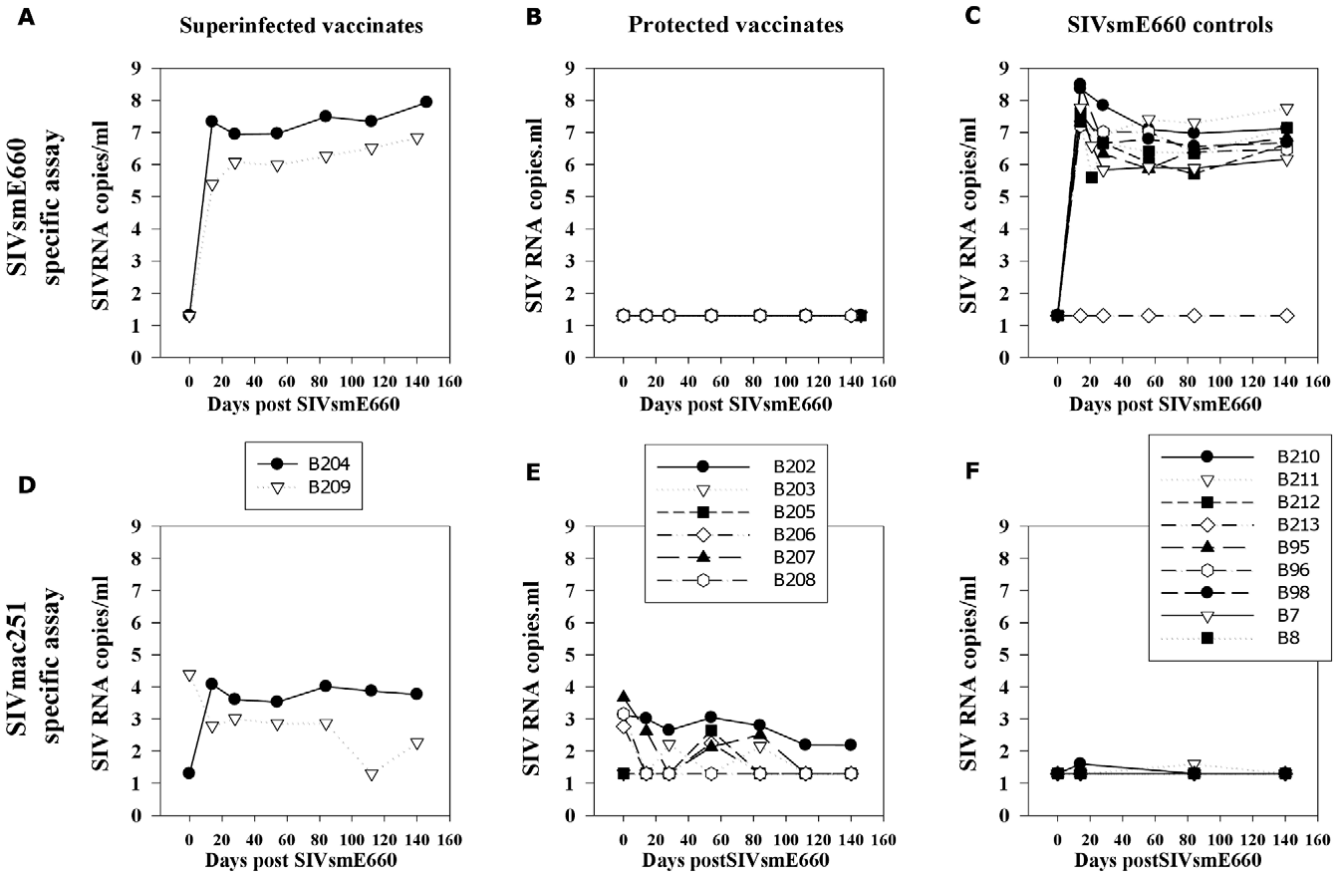
Differential vRNA analysis of SIVsmE660 and SIVmacC8 in plasma. Plasma SIV RNA levels shown for superinfected vaccinates (B204/B209) and six protected vaccinates (B202, B203, B205, B206, B207, B208) using a SIVsmE660-specific viral RNA assay (panels A and B respectively), compared to unvaccinated naive challenge controls (panel C) with the same assay. Controls were eight naïve MCMs challenged with 10 MID_50_ of the SIVsmE660 stock (1/10,000 dilution of the original stock; B7, B8, B95, B96, B210–B213) or productively infected at the 1/100,000 dilution (B98). Levels of the vaccine virus SIVmacC8 are shown in panels D and E for superinfected and protected vaccinates respectively with the SIVmac251-specific qPCR assay [8]. Panel F shows no reactivity >50 SIV RNA copies/ml of the SIVmacC8-specific assay with unvaccinated challenge controls sampled at 14, 84 and 140 days post SIVsmE660 challenge.

**Figure 9:**
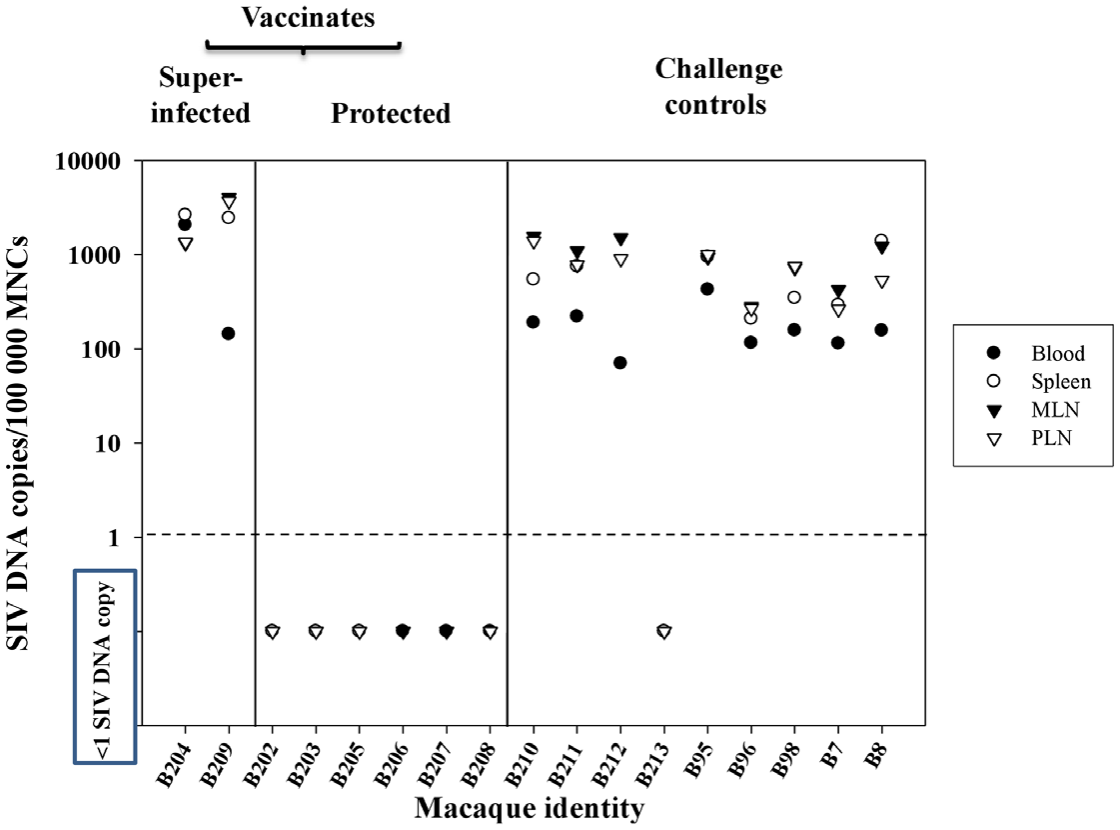
SIVsmE660 DNA levels in lymphoid tissues. Levels of SIVsmE660-specific DNA in lymphoid tissue in SIVsmE660 controls and 20 and 3 week SIVmacC8 vaccinates shown according to superinfection or protection status. SIV DNA levels are expressed as copies of SIVsmE660 DNA / 100,000 cell equivalents MNCs. Limit of detection is 1 copy/100,000 cell equivalents MNC DNA.

Levels of SIVsmE660-specific RNA in plasma and SIVsmE660 DNA (Figures 8 and 9 respectively) in blood and lymphoid tissues in vaccinates (B202–B209) and unvaccinated challenge controls (B210–B213) assessed post-mortem were determined. This identified the ability of vaccination with SIVmacC8 after 3 or 20 weeks to resist SIVsmE660 challenge. Application of the SIVsmE660-specific vRNA assay to all plasma samples collected post-SIVsmE660 challenge separated the vaccinates out into those where vRNA was undetectable (Group A; B202, B203, B205 and Group B; B206, B207, B208), suggesting protection, and those superinfected with SIVsmE660 (B204, Group A; B209, Group B), Figures 8A,B, compared to unvaccinated challenge controls analysed with the same SIVsmE660-specific assay (Figure 8C). No evidence of late breakthrough, or transient spikes in SIVsmE660 RNA signal, was detected in these six protected vaccinates.

These data were supported by analysis of mononuclear cell (MNC) DNA with SIVsmE660-specific qDNA PCR of blood and lymphoid tissues. In 3 out of 4 vaccinates in each group, no evidence of SIVsmE660 DNA was detected in B202, B203, B205 (Group A) or B206, B207, B208, (Group B) at <1 copy SIVsmE660 DNA/100,000 MNC DNA (Figure 9). In superinfected vaccinates B204, B209, high levels of SIVsmE660 DNA were detected at comparable levels to unvaccinated challenge controls (B210, B211 B212, B95, B96, B98, B7, B8) where 100–1000 SIVsmE660 DNA copies/100,000 MNCs were typical. B213 remained SIVsmE660 DNA PCR negative in the tissues sampled. Overall, no evidence of SIVsmE660 sequences were detected in 6/8 SIVmacC8 vaccinates post SIVsmE660 challenge in peripheral blood or lymphoid tissues including spleen, mesenteric and peripheral lymph nodes (MLN, PLN). Interestingly, SIVsmE660 DNA levels in tissues in B98, challenged with 1MID_50_ virus at the infection threshold, exhibited comparable levels of DNA in tissues to the other controls.

### Evidence of vaccine virus recrudescence in vaccinates challenged with SIVsmE660

Vaccine virus recrudescence was detected by the SIVmac251-specific PCR indicative of vaccine re-stimulation in both superinfected and protected vaccinates (Figure 8D,E). However, these were generally at low levels (<1000 SIV RNA copies/ml), shown as a continuum in Figure 3. Lack of reactivity of the SIVmacC8-specific RT-PCR assay at high SIVsmE660 RNA copy number (Figure 8F) confirmed signals detected in vaccinates post-SIVsmE660 challenge to be a true representation of the vaccine virus kinetics. Superinfected macaque B204 exhibited the highest levels of vaccine virus restimulation which also supported the highest level of SIVsmE660 replication post-challenge.

### Distribution of SIV positive cells in lymphoid tissues by in situ hybridisation

**Figure 10:**
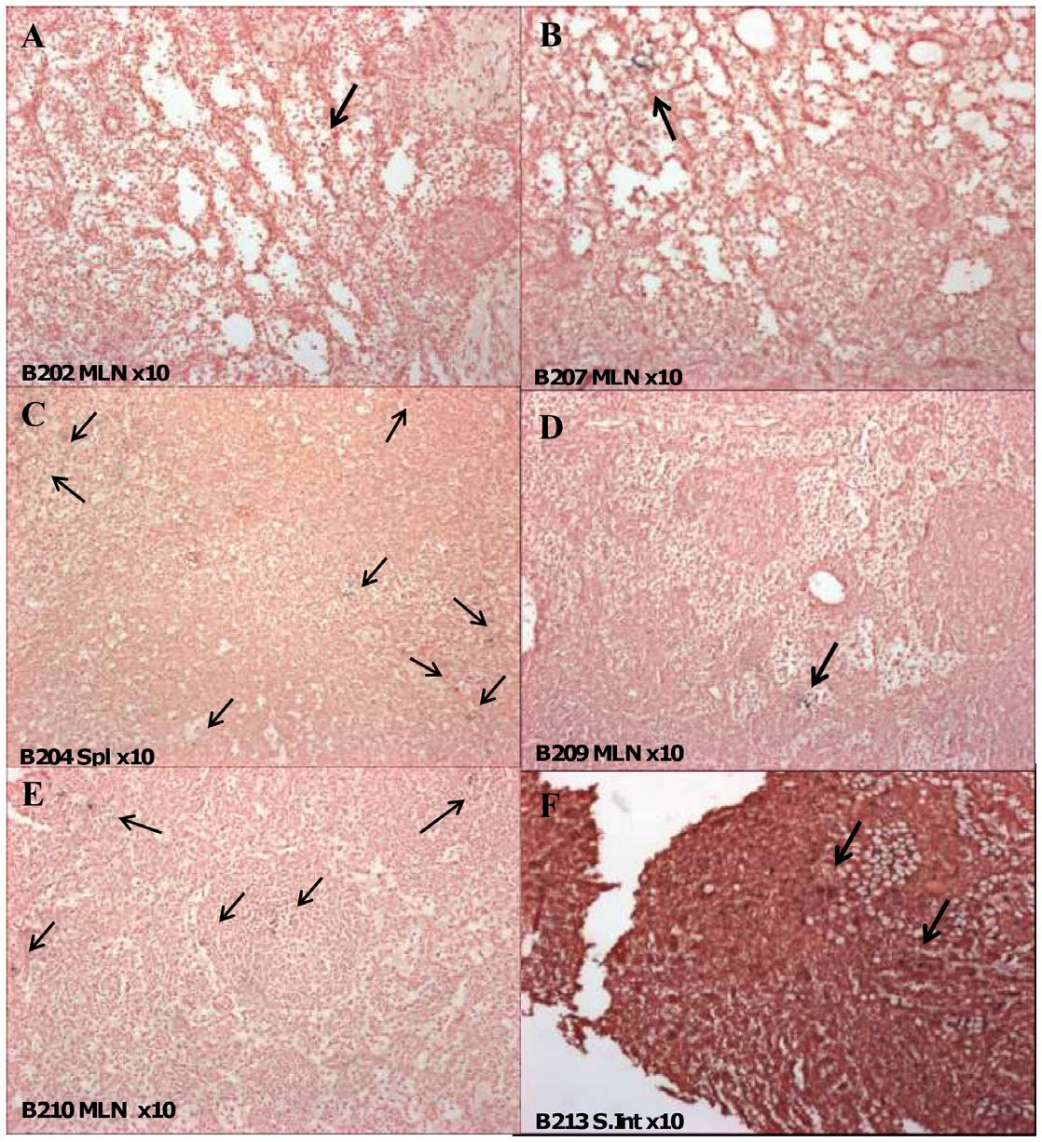
Distribution of SIV RNA positive cells by ISH in protected/unprotected vaccinates and SIVsmE660 controls. Panels A and B) Mesenteric lymph node (MLN) sections from 20 and 3 week SIVmacC8 vaccinates (B202 and B207) protected from SIVsmE660 challenge. Panels C and D show spleen (spl) and MLN from 20 and 3 week SIVmacC8 vaccinates subsequently superinfected with SIVsmE660. B204 shows multiple foci of SIV RNA positive cells in spleen. Panels E and F show MLN and small intestine (S.Int) sections of naive challenge control macaques B210 and B213 after 20 weeks SIVsmE660 infection. B213 exhibits occasional clusters of SIV positive cells. Black staining indicates foci of SIV infection as indicated by arrows.

Distribution of SIV RNA positive cells post-mortem was determined by in situ hybridisation in the vaccine study. Since ISH probe sequences were unable to differentiate between vaccine and challenge virus strains, vaccinates and controls were compared for total SIV RNA. Amongst unvaccinated SIVsmE660 controls challenged for 20 weeks, the frequency of virus-producing cells ranged between 0.5–6.8 SIV RNA positive cells/mm^2^ in most tissues except B210/B212 (MLN) and B212/B213 (spleen) as summarised in Table 3. In the six protected vaccinates at either 40 or 23 weeks post-SIVmacC8 vaccination, SIV positive cells were evenly distributed in all tissues sampled (spleen, MLN, PLN, small intestine, thymus), although frequencies were relatively low (Table 3). Figures 10A and B shows the distribution of SIV RNA positive cells in protected vaccinates B202/B207 (MLN) and a higher frequency of SIV positive cells in superinfected vaccinates B204/B209 (Figure 10C,D). These compare with challenge controls B210 (Figure 10E) and B213 (Figure 10F) for the MLN and small intestine respectively.

**Table 3:** Frequency of SIV RNA positive cells in protected, superinfected vaccinates and challenge controls by in situ hybridisation.

Group	Identity	Vaccine duration	Weeks to ISH	Frequency of SIV ISH +ve cells				
				Spl	PLN	MLN	S.Int	Thy
Protected	B202	20 (A)	40	+	+	+	+	+
vaccinates	B203	20 (A)	40	+	+	+	+	+
	B205	20 (A)	40	+	+	+	+	+
	B206	3 (B)	23	+	+	+	+	+
	B207	3 (B)	23	+	+	+	+	+
	B208	3 (B)	23	+	+	+	+	+
Superinfected	B204	20 (A)	40	+++	+	+	+	+
vaccinates	B209	3 (B)	23	+	+	+	+	+
Challenge	B210	(C)	20	+	+	++	+	+
controls	B211	(C)	20	+	+	+	+	+
	B212	(C)	20	+++	+	++	+	+
	B213	(C)	20	++	+	+	++	+

All vaccinates were challenged with the same dose of SIVsmE660 for the same duration (20 weeks); the total time to sampling is indicated by the vaccination duration of 20 or 3 weeks and group (A–C). The frequency of SIV positive (+ve) cells determined by in situ hybridisation (ISH) is shown for each tissue. Lymphoid tissues sampled were spleen (Spl), peripheral and mesenteric lymph nodes (PLN, MLN), small intestine (S.Int) and thymus (Thy) collected post-mortem. A grading system of + (0.5–6.8), ++ (6.9–13.8) and +++ (>13.8) cells/mm^3^ was employed.

### No impact of MHC genotype on challenge outcome in vaccinates

Of the 12 MCM vaccinated with SIVmacC8 a high frequency (50%) were positive for MHC haplotype M1. Several MCM carried recombinant haplotypes comprising elements of M1 and other haplotypes. B6 was the only individual possessing MHC haplotype M6 in both Class I and Class II regions; B212 was positive for M6 in class IA only. Interestingly, steady-state vRNA loads in control macaque B212 (M6; Class 1A region) challenged with SIVsmE660 was approximately 0.5 log_10_ SIV RNA copies/ml lower than mean values. Of the two vaccinates superinfected with SIVsmE660, B204 was MHC-identical to B5 (M1/M2–M1 recombinant), productively infected in the in vivo titration. An identical genotype to macaque B209 (M3–M1/M3 recombinant) was not present among the other vaccinates though each constituent element of these haplotypes were present in at least one other vaccinate (Figure 2). Finally, the altered infection dynamics in B213 did not appear to be linked to its MHC, since its genotype (M3/M4) was identical to those of B2/B7, both of whom were productively infected with SIVsmE660. B99 (M3/M4), which received less than the minimum dose of SIVsmE660 required to establish infection, remained SIV RNA negative. Overall, no correlation between MHC immunogenotype and outcome of the vaccine study was identified.

### No influence of TRIM5α genotype on vaccine outcome

Similarly, although numbers were too small to apply statistical analysis, there was no overt association with any of the three TRIM5α alleles identified and outcome of the vaccine study. Indeed, the two breakthrough vaccinates (B204, B209) both carried the homozygous Mamu4 genotype [46], as did four other vaccinates evenly distributed between vaccine groups, and eight unvaccinated macaques challenged with varying doses of SIVsmE660 (Table 1). These data indicate there is no apparent confounding factor influencing vaccine outcome relating to TRIM5α polymorphism in this study.

## Discussion

Intensive research efforts to develop a successful prophylactic HIV vaccine have so far proved largely unsuccessful. In macaque models of HIV vaccines, live attenuated SIV elicits robust protection against challenge strains homologous to the vaccine [4], although only limited protection against intravenous challenge with heterologous isolates genetically distinct to the vaccine virus have been reported [29], [18]. Clear correlates of vaccine-induced protection across different studies have proved difficult to identify [7]–[9], [12], [14], [19], [22], [24], [25], [29], [30], hence the immune responses needed to be generated by a successful vaccine remain poorly understood. This study, conducted in Mauritian-origin cynomolgus macaques, describes early potent protection against the heterologous, vigorously replicating SIVsmE660 challenge stock following vaccination with the minimally attenuated SIVmac251/C8 vaccine [35]. Cynomolgus macaques provided consistency with previous studies characterising the protective vaccination conferred by SIVmacC8 [7], [8], [23]–[25].

Mauritian-derived cynomolgus macaques also provided a way to control for host MHC genetics [37]–[45]. Possible confounding non-MHC genetic factors such as TRIM5α polymorphism were also investigated. Against this genetic background the infectious titre and replication kinetics of the SIVsmE660 challenge stock, used without further passage, were determined. In naive MCM, SIVsmE660 established high peak primary viraemia and a higher persisting steady-state infection than reported for Indian RM after delivery by the intravenous route [18], [29], [30]. Productively infected MCM displayed essentially indistinguishable patterns of primary peak and high, stably persisting plasma viraemia, independent of host MHC, TRIM5α polymorphism or challenge dose. Low or limiting challenge doses exhibited comparable plasma viral RNA profiles to higher challenge doses. This level of chronic SIVsmE660 infection initiated persistent depression of CD4 lymphocytes to below the normal range in a proportion of naïve, unvaccinated MCM. Hence, SIVsmE660 provided a rigorous, pathogenic heterologous challenge to evaluate vaccine protection conferred by live attenuated SIVmacC8 in this species, within a defined host genetic background.

Following SIVmacC8 vaccination, a high proportion (6/8) of MCM displayed no evidence of SIVsmE660 superinfection, determined by sensitive and specific real-time PCR assays for SIVsmE660 RNA in peripheral blood and sequestration of SIVsmE660 proviral DNA in lymphoid tissues. No evidence of late breakthrough events in these apparently completely protected vaccinates was identified. One surprising outcome was equivalent levels of protection between 3 and 20 week vaccinates against this heterologous, pathogenic isolate, with one breakthrough case in each vaccine group. Unlike protected vaccinates both exhibited high levels of SIVsmE660 vRNA, comparable to unvaccinated challenge controls, accompanied by a boosting of anti-gp130 titres reflecting increased antigenic stimulation in these cases of viral superinfection. Some immunological benefit, however, was gained from a longer vaccine regime. Profound loss of circulating CD4 lymphocytes in the three week breakthrough vaccinate (B209), comparable to unvaccinated SIVsmE660-infected MCM, contrasted with more preserved CD4 lymphocyte counts in the 20 week breakthrough vaccinate (B204), despite similar levels of SIVsmE660 viraemia.

High efficiency of intravenous transmission with this challenge stock in MCM was further reflected in the ability to successfully infect at the rate limiting 1MID_50_ challenge dose. However, it should be noted that one control macaque in the vaccine study challenged with the 10 MID_50_ dose, failed to develop a productive infection remaining plasma vRNA and proviral DNA negative at all times post-SIVsmE660 inoculation, although did mount a partial anti-gp130 response and SIV RNA positive cells were identified in lymphoid tissue. The reasons for the failure of full virus dissemination in this control macaque are not known, although the host genetic background of this individual did not mark it out from other challenge controls where high, sustained levels of productive infection were typical. Despite this, statistically significant differences between SIVmacC8 vaccinates protected against 10MID_50_ intravenous SIVsmE660 challenge compared to SIVsmE660 controls were observed.

One of the strengths of using Mauritian-derived cynomolgus macaques is the ability to define the haplotype across the entire MHC region encompassing both Class I and Class II, with an association between expression of the Mauritian M6 haplotype and superior control of virus replication following challenge with a stock of SIVmac251 having been demonstrated [44]. However, there was no evidence that possession of any particular haplotype was associated with superior or inferior control of SIVsmE660 replication in unvaccinated MCM or vaccine study outcome. Vaccine breakthrough macaques were not atypical in their overall MHC genetics. Similarly, the MHC genetics of control B213, which exhibited limited replication of SIVsmE660 post-challenge, were not unusual since B2 and B7 possessed the same M3/M4 heterozygous pattern. Hence, the ability of SIVsmE660 to replicate in naive MCM, and subsequent challenge outcome, appears unrelated to the MHC immunogenetic background of this host species.

This would appear to contrast with data from Indian RM where vaccinates expressing MHC-class I alleles associated with control of the vaccine virus also substantially controlled acute phase replication upon SIVsmE660 challenge, although were unable to completely contain SIVsmE660 replication with viraemia levels rising again in the chronic period [18]. One explanation for the lack of association between MHC haplotype and vaccine efficacy in the current study is the limited number in each group (eight vaccinates of which only two were superinfected) which precludes statistical analysis. Another possibility is that the robust replication kinetics of SIVsmE660 in MCM, and the potent protection afforded by SIVmacC8 vaccination, outweigh any advantage that particular MHC haplotypes might confer against SIV infection. Without access to larger cohorts of MCM treated with the same vaccine/challenge virus combinations, detection of robust associations between host MHC genetics, vaccine outcome and viral replication kinetics is statistically challenging, particularly for low-frequency haplotypes.

The relative impact of non-MHC host genetic factors, namely TRIM5α variation was also investigated. In RM TRIM5α is polymorphic impacting to varying degrees on the ability of the host to restrict virus replication in vivo, as recently shown in cohorts of unvaccinated Indian RM inoculated with SIVmac251 [48] and SIVsmE660/E543-related viruses [49]. However, little is known of the impact of TRIM5α genotype on SIV replication in MCM. Sequence analysis of the variable B30.2 domain of TRIM5α identified three distinct TRIM5α genotypes in the 26 MCM in this study, although the Mamu4 allele [46] was present in all 26, with 54% homozygous for Mamu4 and 23% heterozygous for Mafa4/8 and Mafa4/9 respectively. This represents a much more limited spectrum of TRIM5α polymorphism compared to RM and, as with the MHC, most likely reflects the small founder population of Mauritian-origin CM. There appeared to be no impact of TRIM5α genotype on SIVsmE660 kinetics in vivo, nor any confounding impact on the outcome of the vaccine study. However, this does not take into account potential differences in TRIM5α gene expression in vivo in response to vaccination, particularly as differences in vaccine kinetics between vaccinates were noted. More detailed studies would be required to fully resolve this issue. While it is possible that localised expression of anti-retroviral restriction factors may be responsible for the failure of B213 to generate a full infection response, more detailed analyses of the transcriptome profile of this macaque would be required to clarify why disseminated infection did not occur.

The striking observation of this study is the potent protection conferred by SIVmacC8 against SIVsmE660 challenge after only three weeks vaccination, which appears to be unrelated to any confounding host genetic factors. A potent anti-retroviral state appeared to be in place extremely rapidly, irrespective of widely divergent genetic and antigenic variation between vaccine and challenge strains. SIVmac251/C8 displays ∼87%, 83% and 81% nucleotide sequence similarity in gag, env and nef genes respectively to SIVsm-related viruses, comparable to recently described inter-clade differences between SIVmac251 and SIVsmE660 [30]. While these observations do not take into account the biological heterogeneity of different challenge isolates used in challenge studies, the high levels of protection conferred by SIVmacC8 in MCM against the SIVsmE660 stock may be interpreted as representing a strong cross-clade vaccine effect.

This outcome differs from previous studies conducted in Indian RM where vaccination with SIVmacΔ3 [29] or SIVmac239Δnef [18] conferred limited protection against SIVsmE660 challenge. It seems unlikely this could be due to the relative virulence of the viral challenge since SIVsmE660 in MCM establishes a comparably high peak and more persisting viraemia than in Indian RM [18], [29], [30], [50], which is not influenced by host genetics. Vaccine studies with SIVmac239Δnef have also tended to show increasing degrees of protection between 5 and 25 weeks post-vaccination [9], compatible with maturing immunological responses after prolonged vaccination. If differences in outcome are due to the vaccine in the host, vaccination of MCM with SIVmac239Δnef would enable an evaluation of this component of vaccine protection. Characterisation of the vaccine escape viruses in this study at early and late times post-SIVsmE660 challenge may also provide clues as to the genetic composition of the viruses able to evade this otherwise potent protection.

The potential for live attenuated SIV vaccines to achieve such potent heterologous protection needs to be addressed when considering hypotheses of protective mechanisms. Previous studies of protection conferred by SIVmacC8 have not identified a central role for adaptive immune responses detectable in the periphery [24], [25], [8], in agreement with other studies [14], [41]. Superinfection resistance outcomes of reciprocal SIVmac251 and SIVsmE660 heterologous challenges further suggest protection is unlikely to be mediated by peripheral adaptive immune responses [30]. Recent studies conducted in MCM, however, while further confirming high frequencies of functional CD8+ T-cell responses are not induced in the peripheral blood after live attenuated SIV vaccination, have detected CD8+ T cells recovered from mucosal tissues, such as the lung, capable of suppressing virus after only 8 days [41]. Unfortunately, the design of the current study precluded a formal investigation of mucosal-based immunity. Clearly, whether the protective processes are the same for low-dose mucosal SIVsmE660 challenge in this species would be an important question to address.

However, our recent analyses of primary SIVmacC8 infection focussing on early events in the gut mucosae, a primary site of HIV/SIV replication [51]–[55], have shown the replication kinetics of SIVmacC8 in MCM to have a profound impact on immune cell population dynamics post-inoculation [56]. Intestinal lymphoid cells expressing CD4+/CCR5+ receptors markedly depleted following inoculation of SIVmacC8 at early times recover by 20 weeks, suggesting target cell depletion per se cannot account for later protection [56]. In the RM model, maintenance of the intestinal CD4+ memory T cell population has been associated with vaccine protection independent of clear immune correlates of protection [17]. Unravelling the relative contribution of early changes in target cell dynamics in the gut mucosae and long term changes, perhaps driven by persistence of the vaccine virus across a wide number of body lymphoid compartments, will be important to assess early and late vaccine responses. Differences in levels of CCR5 expression between different species may also be a factor when interpreting inter-species differences in outcome [56].

One consistent feature of live attenuated SIV vaccine studies is that protection appears inversely proportional to the degree of viral attenuation [13], more vigorous and persisting vaccine viruses associated with higher levels of protection. Conversely, highly attenuated viruses make very poor vaccines [57], with some SHIV-based vaccines conferring more limited vaccine protection over extended studies [58], [59]. In this study, in protected vaccinates SIVmacC8 persisted in a wide range of lymphoid tissues sampled post-mortem including the spleen, peripheral and mesenteric lymph nodes, small intestine and thymus indicated by a consistent frequency of SIV RNA positive cells, widely distributed among lymphoid organs. This characteristic feature of SIVmacC8 vaccination [8], [10] may contribute to its ability to resist a range of challenge viruses via different routes.

A direct role for an actively replicating retrovirus in live attenuated SIV vaccine studies seems further supported by the fact that protection against re-challenge can be associated with significant re-stimulation of the vaccine virus [8], [21]. Although the levels of vaccine virus recrudescence detected in protected vaccinates in this study were lower than previously reported when re-challenged with a vigorously replicating homologous wild-type virus [8]. Interestingly, vaccine virus re-stimulation was highest in vaccinate B204 where simultaneous replication of the superinfecting SIVsmE660 virus and co-stimulated vaccine virus were detected together. Whether this is related to the more controlled vRNA kinetics in the 20 weeks SIVmacC8 vaccination prior to SIVsmE660 challenge is unclear (see Figure 3), but indicates altered infection dynamics during the vaccination period in this vaccinate. New vaccine tools, such as the conditionally live attenuated SIVrtTA vaccine [60] where the kinetics of the vaccine virus may be modulated, may provide further insight into the role of vaccine persistence.

Concepts of vaccine protection centred around target cell population dynamics, vaccine persistence and cell permissivity post-vaccination focussed around localised vaccine-induced responses could account for the apparent paradox of superior protection against heterologous virus challenge (SIVsmE660) compared to more limited early protection against vigorous, homologous wild-type virus challenge (SIVmac251/32H/L28 stock) [8]. Analysis of the early pathogenesis of these two distinct virus stocks in vivo will further address this, since localisation of virus by ISH suggests an altered distribution of virus between protected vaccinates and challenge controls. Induction of localised innate immune responses, perhaps driven by persisting SIV replication at key sites of infection may also influence direct viral competition [61]. The ability to derive potent protection against SIVsmE660 as soon as 3 weeks post SIVmacC8 vaccination augments earlier observations where time to protection studies conferred by SIVmacC8 against wild-type virus challenge have demonstrated an early protective effect in this model system, which is partially protective as early as 10 days post-vaccination [24]. As adaptive immune responses have so far not been found to be central to this protection, we are considering whether innate immune responses may be at play. This model will enable evaluation of whether an up-regulation of interferon-inducible anti-retroviral restriction factors [62], [63] which, for example, may be contributing to a state of retroviral superinfection resistance in vivo or whether some other anti-viral interference mechanism is responsible for the protection observed. Determining the relative contributions of innate and adaptive immune responses in this mode of vaccine protection will be important to better our understanding of this potent vaccine approach.

In summary, early, potent protection against a vigorously replicating, heterologous viral challenge (SIVsmE660) was demonstrated in Mauritian-origin cynomolgus macaques. Using this genetically characterised model, potent vaccine protection was generated by administration of a persisting live attenuated SIV for three weeks. Defining how this works will inform the field whether it can be harnessed to aid development of effective AIDS vaccines for clinical use.

## Materials and Methods

### Virus stocks

The SIVsmE660 challenge stock was obtained courtesy of Dr. Vanessa Hirsch and Dr. Philip Johnson through the AIDS Research and Reference Reagent Program, Division of AIDS, NIAID, NIH, USA, originally derived from spleen cells of a rhesus macaque inoculated with blood from RhE543, a SIVsmF236-infected rhesus macaque [64]. SIVsmE660 was selected since it represents a pathogenic, uncloned virus stock, considered to be a heterologous isolate with respect to SIVmac239/SIVmac251 viruses which replicates in lymphocytes and macrophages. Independent titration experiments with this stock were performed to determine the infectivity and end-point titre of the SIVsmE660 challenge stock in naïve cynomolgus macaques, used directly as supplied, with no further passage or adaptation in cynomolgus macaques or cynomolgus PBMCs in vitro.

The vaccine virus, SIVmacC8, is a virus clone characterised to have an attenuated phenotype in vivo, the result of a 12bp (4 amino acid) in-frame deletion in nef and two additional conservative amino acid changes [35]. In the vaccine study, macaques were inoculated intravenously with 5000 TCID_50_ SIVmacC8 (9/90 pool).

### Ethics statement

The non-human primates in this study were used in strict accordance with UK Home Office guidelines. The work at NIBSC was governed by the Animals (Scientific Procedures) Act 1986 which complies with the EC Directive 86/609. The work was performed under licence PPL 80/1952 which was granted only after review of all the procedures in the licence by the local Ethical Review Process (ERP) at NIBSC.

All individuals in the study were purpose bred and group housed for the entire duration of the study. Regular modifications to the housing area including the introduction of novel structures and the introduction of foodstuffs in novel manners were made by husbandry staff to enrich the environment during the study. All animals were sedated prior to bleeding or inoculation of virus by venepuncture. Regular, frequent checks were made by staff and any unexpected change in behaviour by individuals on study followed up, including seeking of veterinary advice where necessary. Regular blood samples were obtained to assess haematological parameters in blood that might provide evidence of incipient disease and veterinary advice was sought when persisting abnormalities detected. The study was terminated and all animals killed humanely by administering an overdose of anaesthetic prior to the development of overt symptoms of disease. All efforts were made to minimise suffering.

### Experimental outline

Twenty-six naïve, simian D-type retrovirus free, juvenile Mauritian cynomolgus macaques (Macaca fascicularis) were used. In vivo titration of the SIVsmE660 stock was performed in two series by intravenous inoculation of a total of 14 naive MCM. Dilutions of the SIVsmE660 challenge stock were prepared directly from the NIH stock, without any onward passage, in RPMI 1640 media, in ten-fold steps. Initially, four groups of two macaques were used (Group 1, B1, B2; Group 2, B3, B4; Group 3, B5, B6; Group 4, B7, B8) bled at 0, 7, 10, 14, 21, 28, 54, 86 and 140 days post-infection (p.i). and a further six to establish the final end-point (Group 5, B95, B96; Group 6, B97, B98; Group 7, B99, B100) bled at 14, 28, 54, 86 and 140 days p.i.

The vaccine study comprised three groups of four MCM, vaccinated with 5000 TCID_50_ SIVmacC8 [35], either for 20 weeks (B202–B205; Group A) or 3 weeks (B206–B209; Group B). Twenty week vaccinates were bled at intervals post-SIVmacC8 inoculation (0, 14, 21, 56 and 140 days) and 3 week vaccinates at 0 and 21 days post-vaccination. All vaccinates were challenged with 10 MID_50_ (MID_50_ =  macaque infectious dose where 50% of macaques are infected) of SIVsmE660 challenge stock with four additional naïve challenge controls (B210–B213; Group C) in the vaccine study bled at 0, 14, 28, 54, 84, 112 days post-SIVsmE660 challenge, euthanased humanely at ∼140 days post-infection.

### Real time QPCR to quantify total SIV RNA levels in plasma

Total plasma SIV RNA levels in naive MCM challenged with dilutions of the NIH SIVsmE660 challenge stock in the in vivo titration were assessed by quantitative real-time PCR. Using conserved sequences located in the R/U5 region of genomic SIV RNA, an assay was developed using a previously validated SIV RNA reference panel [65]. Viral RNA was extracted from 140 µl plasma using viral RNA mini-kits (QIAamp; Qiagen) according to the manufacturer's instructions, eluted in a final volume of 50 µl AVE elution buffer. RNA (5 µl) extracted from reference or experimental samples were amplified, in triplicate, using UltraSense one-step RT-PCR kits (Invitrogen Ltd). The R/U5 qRT-PCR assay was performed with forward primer**:** (5′-CTCCACGCTTGCTTGCTTAA-3′), reverse primer (5′-AGGGTCCTAACAGACCAGGG-3′) and Taqman hydrolysis probe sequence (5′-6′-TCCCATATCTCTCCTAGYCGCCGC-3′-BHQ1). Optimised thermal cycling profiles were 51C/30mins for the RT step, inactivation/activation step at 95C/10 mins, and 45 cycles of denaturation (95C/30sec), and annealing/elongation (60C/90sec) performed on an Mx3000P genetic analyser (Stratagene Ltd). Quantitative data were determined using the Mx3000P software. The assay had a lower limit of detection of 50 SIV RNA copies/ml plasma.

### Amplification and sequencing of SIV gag

PCR amplification of ∼700bp region of SIV gag was performed on DNA template derived from unvaccinated macaques challenged with the SIVsmE660 challenge stock using nested PCR primers: forward/outer (5′-ATGGGCGCGAGAAACTCCGTC-3′) reverse/outer (5′-CTACTGGTCTCCTCCAAAGAG-3′) forward/inner (5′-AACAAGTAGACCAACAGCAC-3′) reverse, inner (5′-TCCCCTCTGTTGGACTGCT-3′). Amplification products were diluted 1∶15 and sequenced as previously described [8]. Sequences were assembled, aligned and compared with published database sequences for SIVmac/sm strains (www.hiv.lanl.gov).

### Detection and differentiation of SIVmacC8 and SIVsmE660 by PCR

Real-time PCR assays which differentiate between the vaccine (SIVmacC8) and challenge (SIVsmE660) viruses were applied to plasma and PBMC purified from peripheral blood and mononuclear cells (MNCs) derived from lymphoid tissues (spleen, MLN, PLN) collected post-mortem. SIVmacC8 was detected using the gag assay as previously described [8]. An analogous assay was developed by modification of primers to detect SIVsmE660 sequences, but not SIVmac251/C8.

Optimised SIVsmE660-specific primer/probe combinations in gag yielded a 75bp amplicon: 50nm SIVsmE660-specific forward primer (5′-GCTGCCGATTGGGATTTACA-3′), 900nM SIVsmE660-specific reverse primer (5′-GTCTGATCCTCTTGGCTCTCTAAGTT-3′); 75nM SIVsmE660 specific probe sequence (5′FAM-CGCAGCCAGGTCCACTACCAGCA-3′-BHQ1). Amplifications for vRNA analysis were performed using Ultrasense one-step RT-PCR kits (Invitrogen) with 5 µl RNA. Cycling conditions were 30sec/54C (reverse transcription), hot start 10min/95C and a two-step amplification of 30sec/95C and 90sec/60C for 45 cycles performed on a Stratagene Mx3005P thermal cycler.

### Virological and serological assays

Virus isolation was performed on Ficoll-purified PBMC following 28 days co-culture with C8166 indicator cells, evidence of virus replication by syncitium formation and p27 antigen detection [66]. Anti-gp130 levels were determined by enzyme immunoassay [25], with SIV envelope rgp130 (EVA670/CFAR/NIBSC) antigen. Neutralizing antibody titres were determined by mixing serum serially diluted in RPMI containing 10% FCS with virus. Serum dilutions representing 75% inhibition p27 antigen production represented the titre. SIVsmE660 challenge stock was used directly to assess neutralising antibodies against the challenge virus and SIVmac251/J5 to represent the vaccine virus.

### Immunological and haematological analyses

CD3+/CD4+ and CD3+/CD8+ lymphocyte populations were monitored in whole blood by flow cytometry following immunostaining with cross-reactive anti-human monoclonal antibodies. Whole blood (200 µl) was incubated with 10 µl of fluorescein isothiocyanate (FITC)-labelled anti-monkey CD3 monoclonal antibody, FN18 (Serotec), 20 µl of phycoerythrin (PE)-labelled anti-human CD4 monoclonal antibody, Leu-3a (BD Biosciences), 10 µl of APC-labelled anti-human CD8 monoclonal antibody, 3B5 (Caltag), 1hr 4C. FACS lysing solution (BD Biosciences) was used to remove red blood cells; cells were washed twice (PBS containing 4% (v/v) FCS/0.1% sodium azide). Samples were analysed by FACS (FACSCalibur, BD Biosciences), after fixing cells overnight in 2% (v/v) formaldehyde in PBS, gating on the lymphocyte fraction.

### In situ hybridization analyses

In situ hybridisation (ISH) was performed on lymphoid tissues (spleen, MLN, PLN, small intestine, thymus) collected post-mortem as previously described [67]. Hybridisation mixes consisted of sense or anti-sense probes to SIV gag, env and nef transcripts. Quantitative ISH data were determined by manually counting positive cells within up to 10 random fields of view (x10 lens and x10 eye-piece magnification; equivalent to an area of 2.2mm^2^). The mean number of positive cells/mm^2^ was expressed using a grading key (see Table 3).

### MHC class IA and IB and II haplotype characterisation of Mauritian cynomolgus macaques

MHC class IA and IB and class II haplotypes in Mauritian cynomolgus macaques were determined by microsatellite PCR with resolution of recombinant haplotypes by allele-specific PCR as previously described [44].

### Sequence analysis of TRIM5α

An 847-bp TRIM5α gene fragment encompassing the B30.2 domain (exon 8) was amplified from 200 ng genomic DNA in 25 µl reactions comprising: 10 mM Tris-HCl, pH 8.3; 50 mM KCl; 2 mM MgCl_2_; 0.5 µM of each oligonucleotide (TRIM5-Ex8-S, 5′-GTA AGG AGA AGT CAC ATT ATC A- 3′ and TRIM5-Ex8-A, 5′-TCA AGA GCT TGG TGA GCA CAG-3′); 0.2 mM each of dATP, dCTP, dGTP and dTTP and 1.25 U AmpliTaq Gold DNA polymerase (Applied Biosystems, USA). Amplification consisted of a single cycle at 94C/2 min, followed by 39 cycles comprising 94C/30 sec, 64C/45 sec, 72C/2 min and a single 5 min incubation at 72C. For each sample two independently amplified fragments were bi-directionally sequenced using BigDye Terminator v3.1 cycle sequencing (Applied Biosystems, Cheshire, UK). Sequences were aligned and analysed using *MEGA* Version 4 [68].

### Statistical analyses

Data were presented graphically using Sigma Plot version 8.0 (SPSS Inc). Standard error determinations of mean (± SE) viral RNA copy number and comparison of plasma viral load (SIV RNA copies/ml) between vaccinated and naïve challenge macaques by nonparametric Mann-Whitney *t* test, were performed using MiniTab (version 15) software.

## Supporting Information

Table S1 **Concordant outcome in productive infection by vRNA and virus isolation in MCM challenged with serial dilutions of the SIVsmE660 challenge stock.** Nt  =  not tested. The number of infected macaques is shown for each pair of macaques challenged. vRNA  =  viral RNA; VI  =  virus isolation.
(PDF)
