# Supplementary material for: Early Potent Protection against Heterologous SIVsmE660 Challenge Following Live Attenuated SIV Vaccination in Mauritian Cynomolgus Macaques
Source: PLoS One. 2011 Aug 10;6(8):e23092. doi: 10.1371/journal.pone.0023092 (PMC3154277; doi:10.1371/journal.pone.0023092)
Supplement: Table S1 — Concordant outcome in productive infection by vRNA and virus isolation in MCM challenged with serial dilutions of the SIVsmE660 challenge stock. Nt = not tested. The number of infected macaques is shown for each pair of macaques challenged. vRNA = viral RNA; VI = virus isolation. (PDF) [file pone.0023092.s001.pdf]

| Macaque | Reciprocal dilution SIVsmE660 | vRNA ($log_{10}$) SIV RNA c/ml | | VI | Number infected |
|---|---|---|---|---|---|
| | | Day | | | |
| | | **10** | **14** | **14** | |
| **B1** | 10 | 7.49 | 6.58 | + | 2/2 |
| **B2** | 10 | 7.39 | 6.29 | + | |
| **B3** | 100 | 7.60 | 6.82 | + | 2/2 |
| **B4** | 100 | 7.43 | 6.69 | + | |
| **B5** | 1,000 | 7.60 | 7.70 | + | 2/2 |
| **B6** | 1,000 | 7.31 | 6.30 | + | |
| **B7** | 10,000 | 5.72 | 7.15 | + | 2/2 |
| **B8** | 10,000 | 5.63 | 6.98 | + | |
| **B95** | 10,000 | nt | 6.93 | + | 2/2 |
| **B96** | 10,000 | nt | 7.03 | + | |
| **B97** | 100,000 | nt | <1.30 | - | 1/2 |
| **B98** | 100,000 | nt | 7.61 | + | |
| **B99** | 1,000,000 | nt | <1.30 | - | 0/2 |
| **B100** | 1,000,000 | nt | <1.30 | - | |
